# Organization of professional practices against intrafamily violence against children and adolescents in the institutional context**^1^**

**DOI:** 10.1590/1518-8345.1640.2889

**Published:** 2017-06-05

**Authors:** Gabriele Schek, Mara Regina Santos da Silva, Carl Lacharité, Maria Emília Nunes Bueno

**Affiliations:** 2 Doctoral student, Universidade Federal do Rio Grande, Rio Grande, RS, Brazil. Scholarship holder at Coordenação de Aperfeiçoamento de Pessoal de Nível Superior (CAPES), Brazil.; 3 PhD, Associate Professor, Escola de Enfermagem, Universidade Federal do Rio Grande, Rio Grande, RS, Brazil.; 4 PhD, Full Professor, Département de Psychologie, Université du Quebec à Trois-Rivières, Trois-Rivières, QC, Canada.; 5 PhD, RN, Associação de Caridade Santa Casa do Rio Grande, Rio Grande, RS, Brazil.

**Keywords:** Domestic Violence, Child, Adolescent, Professional Practice, Institutional Organization

## Abstract

**Objective:**

to analyze based on the practitioners' discourse, the way they organize their practices confronting situations of intra-family violence against children and adolescents.

**Method:**

qualitative research carried out with 15 professionals who work in social and health services located in the southernmost of Brazil. Data were collected through semi-structured interviews, performed at the participants' workplace. We used a theoretical matrix to analyze the data, based on Institutional Ethnography and the technique of discursive textual analysis.

**Results:**

the practitioners' practices developed in situations of intra-family violence against children and adolescents are organized on the basis of: power relations that take place in services that respond to violence situations; routines instituted to meet the demands of care in services; and the interplay between the conception of violence as a public health problem and the conception of violence as a social problem.

**Conclusion:**

the way these practices are organized is reflected in actions that are not protective against situations of intra-family violence against children and adolescents.

## Introduction

Strategies for intervention in situations of domestic violence against children and adolescents have been expressed in some official documents, among them the Statute of the Child and Adolescent (ECA) and the Primary Health Care Handbook - Intra-family violence against children and adolescents: guidelines for practice in service. The first document places Brazil in a prominent position in the world scenario for establishing a law considered one of the most advanced in terms of guaranteeing the rights of the child and youth population^1^; The second aims to support states and municipalities in the implementation of actions that promote equality and the exercise of human rights^2^.

From what these documents propose, professionals have the responsibility to identify victims in an early stage, to notify suspected or confirmed cases of violence^1^, and, to promote actions to strengthen relations between institutions working in the areas of health, safety, justice, education, human rights and social movements, aiming to guarantee adequate protection and treatment for children and adolescents^2^.

Although these documents point out guidelines for organizing effective actions in terms of protecting victims of domestic violence, according to the World Health Organization (WHO) it is necessary to consider among others, the low participation of professionals in complaints and notification of situations of violence^3^, contributing to the fact that many cases remain anonymous. This leads to questions about the difficulties that professionals experience in incorporating in their practice the guidelines for the protection of children and adolescents as described in the official documents and serving as foundations to organize their practices in the face of situations of intra-family violence, present in their daily routine in the health services in which they operate.

The literature suggests that the professional practices developed in the health services are based on power relations that hinder the integration between professions and professionals^4^. As a consequence, the practices developed in the context of these services tend to be fragmented and individualized, proving to be ineffective in the face of the complexity of the problem, which can not be understood by individual competence in a single professional area, but rather involving multidisciplinary and intersectoral actions ^5^.

In addition, professionals rely on pre-established norms and routines that tend to situate them in certain spaces and to regulate their daily practices^6^. On the other hand, the lack of emotional support to deal with situations of intra-family violence against children and adolescents contributes to the failure of many professionals to assume their responsibilities, thus transferring the situations to other services^7^.

In order to understand how the organization of practices occurs in the face of situations of intra-family violence, it is fundamental to give voice to practitioners working in this context, understanding the phenomenon of violence based on their experiences and the problems they experience in the daily life of their work, and in the dominant institutional and social relationships that shape, limit and organize their practices.

In view of the above, this study aimed to analyze, based on the practitioners' discourse, how the organization of their practices confronting situations of intra-family violence against children and adolescents.

## Method

This is a qualitative study carried out with 15 professionals, six nurses, two psychologists, two physicians, two community health agents, two guardianship counselors and a social worker, who met the following inclusion criteria: have had under their care children and adolescents attended as a result of intra-family violence, (being it presumed or confirmed) and to be linked to the service where they have been for at least 12 months.

The professionals were recruited in services that serve children and adolescents victims of intrafamily violence, including Emergency Care Units and Pediatrics of a University Hospital, a Basic Health Unit, a Specialized Referral Center in Social Assistance (CREAS) and a Guardianship Council, based in a medium-sized municipality, located in the southernmost of Brazil. The option to include these services is due to the fact that, as a whole, they portray the itinerary usually traversed by families in situations of violence in the municipality.

The University Hospital serves the population free of charge through the Brazilian National Health System (SUS). It has 189 beds for hospitalization, distributed in different purposes, and two units stand out in regards to the care of children and adolescents: the Emergency Care Unit (UPA), which serves approximately thirty children and adolescents daily and the Unit of Pediatrics, with the capacity to hospitalize about twenty patients.

The Basic Health Unit is composed of three Family Health teams, made up of doctors, nurses, nursing technicians and community health agents. Each of the teams serves approximately 4,500 families, with the following responsibilities: welcoming users, facilitating access to other services that make up the network and implementing healthcare activities, prioritizing individuals, families and groups with greater risk and vulnerability.

The Guardianship Council is a permanent, autonomous, non-jurisdictional public body charged by society to ensure the rights of children and adolescents. Among its attributions are the attendance to situations that involve threat or violation of the rights of the child or adolescent; application of protective measures; care and counseling of parents or guardians; requesting services and making referrals to the service network. This institution has twenty counselors distributed in three teams, responsible for the annual care of approximately one thousand children and adolescents victims of intra-family violence.

CREAS has the responsibility of providing specialized assistance to individuals or families in situations of personal or social risk, for violation of rights. Weekly, it attends about thirty cases involving intra-family violence against children and adolescents. Several agencies such as the Guardianship Council, Police Station and Juvenile Court and Youth refer 95% of these patients.

Data were collected between November 2013 and March 2015, through semi-structured interviews conducted individually and in the work place of the professionals. These interviews were guided by a script that included the investigation of professionals' knowledge regarding official documents that refer to intra-family violence; the institutional routines adopted to attend to these situations and the facilities and difficulties to develop protective actions in the institutional context. Each of the interviews lasted, on average, an hour and 15 minutes.

To preserve anonymity, the professionals were identified by the letter P, followed by the word "health" or "social", corresponding to the area of performance of each of the professionals interviewed. Example: (P_health_), (P_social_)_._ This study was approved by the Research Ethics Committee of the institution to which it is affiliated, with registration under the number 066/13.

For the organization, analysis and interpretation of the data, a theoretical matrix was built based on Institutional Ethnography (IE). The IE considers that the social relations and the ways work is established in institutional contexts are mediated by "texts", which are considered the main elements of coordination and regulation of the work activities^8^. In this study, the "texts" are represented by public policies, institutional routines, power relations and by some forms of discourse that permeate the institutional context.

During the process of analysis, the political and bureaucratic interests that "texts" can carry^8^, producing standardized methods of work that exclude the point of view of people and contribute to the production of automatic and non-reflexive practices in certain situations^9^.

The technique of analysis was the textual discourse^10^, following the steps: dismantling the interviews, identifying the institutional forces present in the discourse of professionals and forming the units of analysis; then build relations between these units, grouping their elements into a process that resulted in three categories: relations of power as mediators of professional practices; institutional routines and the need to meet the demands; family violence against children and adolescents: a social or health problem?

## Results

### Characterization of participants

Among the 15 participants in the study, 14 were female and one male, aged 28-64 years. The tenure time varied between 12 and 240 months. Twelve of the professionals have complementary training, with special emphasis in the areas of Public Health and Family Health.

Of the six nurses who participated in this study, three work in an University Hospital, in the areas of pediatrics and Emergency Care, and three integrate the Family Health teams of a Basic Health Unit located in the periphery of the municipality. The two physicians and two community health agents are linked to the same Basic Unit. The two guardianship counselors interviewed are from different teams. Two psychologists and a social worker develop their professional activities in a Specialized Reference Center on Social Assistance.

### Power relations as mediators of professional practices

The practitioners' discourse highlighted the power relations that permeate the context of the services where they work and that influence the way in which they organize their practices when confronting intrafamily violence situations. These power relations were more prominent among physicians and nurses, and nurses reported that sometimes their care for victims and families is interrupted due to the decisions of physicians. *There are doctors who think they know everything. One time I was talking to a mother, trying to figure out some things, because I suspected of a situation of negligence. Then I got a call from the doctor saying that I was supposed to pass the appointment right away, because I was taking too long. I may have taken long, so I ended up complying with it and immediately forwarding the child to the doctor's office.* (P_health_)

The power relations were also evidenced between the Guardianship Council, the Hospital and the Basic Health Unit. The professionals reported that there are communication bottlenecks between institutions, a predominance of the decisions of the Guardianship Council regarding the outcomes of intra-family violence cases, even when the evaluation of a given situation differs among professionals. *A colleague and I were accompanying a woman who was pregnant for the fifth time. The children lived in complete neglect and anyone could see that. This situation was referred to the Guardianship Council and soon after it was closed. My colleague was called by the Council to hear from them that we were wrong. However, they never called us to discuss the situation, to understand the reasons that led us to make the referral. And it was like this: case closed.* (P_health_)

The professionals who work at CREAS testify that one of the main difficulties encountered in dealing with situations of intra-family violence against children and adolescents is the bureaucracy and the demands imposed by the judiciary. Among them is the production of medical reports and reports used in legal proceedings. These professionals recognize that they must act jointly before the courts, however, they affirm that the determinations of the judicial services impose changes in their routines that are directly reflected in the assistance provided to children and adolescents victims of intra-family violence. *The role of CREAS today is not what we produce. What we do are reports for the justice, because that is the great demand upon us. It was for the judiciary to have a wellpaid professional to do that. Since here in the city we do not have one, it falls on us who have to make these reports, because, according to them, if we do not do them we can be accused of obstructing justice.* (P_social_)

### Institutional routines and the need to meet the demands

In the professionals' discourse it was possible to identify concerns in terms of supply and demand in relation to social and health services. The municipality in which this study was developed is located in one of the main naval hubs of Brazil, which in recent years has caused intense migration to the region causing socioeconomic impact and causing changes in the organization of services, given the need to care for the entire population.

One of the interviewees stated that institutional routines are now determined by the supply and demand of services: *The population of the municipality has increased greatly in recent years and violence has also increased. We still continue with two hospitals, that is, in the past I served 40 people in six hours of work, today I need to attend on average 80 to 85 people. I calculate that I have about five minutes to attend a person and I know that in situations of violence that time is insufficient.* (P_health_)

CREAS professionals also highlighted the readjustment of their reception routines, motivated not by the need that the families presented, but due to the increase in the demand for monthly care: *Depending on the number of monthly visits, we organize ourselves as follows: we hold four sessions, individually with the children who arrive at the service to obtain a diagnosis. If necessary, we refer them to the follow-up groups, which last 12 weeks. I do not know if it is enough for these situations, but this is what we can do.* (P_social_)

With the evaluation of many children and adolescents, the need for individualized treatment was verified. However, there are insufficient the services in the municipality that provide this type of care to the victims. This situation represents one of the main challenges encountered in the practice of these professionals. *We evaluate the situation, but then what? Many cases are referred to the Family Health Support Center (NASF), but this service has only one psychologist who serves ten teams. Each team serves about 30 thousand families. How will she do the service? This is impossible.* (P_health_)

### Intra-family violence against children and adolescents: a social or health problem?

This last category highlights the interplay between the conception of violence as a public health problem and violence as a social problem. To P_(social)_, the SUS understands intra-family violence as a social problem; however, the Unified System of Social Assistance (SUAS) considers that treating the victims is a task of the health services. Thus, responsibility is not assumed by any of them. *We of social assistance provide what SUAS calls psychological support to victims and families to help in the recovery of the bonds. This is not treatment. There are children who need individual treatment. Well, now we are talking about health, and this type of intervention is not included in the SUS. Here in the municipality, we only have the Child Psychosocial Care Center, but it only tends to more serious situations. So, who treats this victim?* (P_social_)

From the perspective of some participants there is a lack of consensus regarding the responsibilities of each service that makes up the network of protection for children and adolescents. This contributes to make many families to go through a long journey in the search for care.

## Discussion

Professionals who work in care services for children and adolescents who are victims of intra-family violence need to make decisions that will impact the life and development of these individuals. Considering this factor, a study of 828 protection-services professionals from four countries (Israel, the Netherlands, Northern Ireland and Spain) revealed the importance of an institutional organization in decision-making and victim assistance interventions. Mutual cooperation between practitioners and institutions has proved to be one of the main tools for the adoption of efficient protective practices^4^.

Differently from the results of the aforementioned research, the data of this study showed that, in the different institutional contexts investigated are permeated by power relations that coordinate, limit and organize the professional practices developed in these spaces. In addition, there was the establishment of institutional routines defined mainly for institutional demands, neglecting the needs presented by the victims and families.

With regard to power relations, these were evidenced as mediators in the organization of practitioners' practices, especially among doctors / nurses, guardianship counselors / social and health workers, and judicial servants / CREAS members. Power relations were also evidenced in a study carried out with nurses, in which power-determining factors such as the qualification of positions and their level of social prestige were identified. These relationships can be one of the main causes of the conflicts that are established between the professionals, generating stress, dissatisfaction in the work and low effectiveness in the performance of the team^11^.

In view of this, there is a need to further problematize the power relations that are established in the context of institutions, since they contribute to the fragmentation of care, compromising the assistance provided to users^12^. For some authors, work practices aimed at protecting children and adolescents should efficiently articulate the different sectors that make up the protection network, integrating them both in macro-structural (political) aspects and in internal aspects of services, seeking inter-institutional and interpersonal interactions^13^.

The power relations identified in the scope of the Guardianship Council may be related to the social role of this institution, insofar as this Council is seen as a reference body, charged by society to ensure the fulfillment of the rights of children and adolescents, as described in the ECA^1^. However, it is necessary to question whether such power relations, reported by some professionals, do not constitute an alibi for a less participative action of the other professionals in the intervention processes.

In this sense, a study developed with tutorial advisers emphasized that social and health institutions do not monitor the protective measures taken in the scope of the Guardianship Council. As a result, many cases have ceased to be monitored and are eventually lost. Thus, the importance of better communication between the institutions, with a view to promoting collective actions, controlling workflows and monitoring these situations is evident.^13^.

For some authors, regardless of the different professions involved in the protection of children and adolescents, there is a need for professionals to establish and share responsibilities for situations dealt with in the daily work situation, providing a more accurate evaluation of the situation and a follow-up and treatment that addresses the needs of victims and families^14^. In addition, the authors pointed out that the sharing of responsibilities among professionals is an efficient way of reducing negative factors such as personal prejudices and subjectivity in decision making^15^.

It was also verified that the organization of professional practices, in the face of situations of intra-family violence was originally organized based on institutional routines specially formulated to meet the high demand for care, thus filling the deficits of services offered by the municipality. This form of organization is also mentioned in a research that explored some of the obstacles encountered by professionals regarding the care of women victims of violence in the context of public health services. These services were subordinated to a series of norms and routines, among them, the accomplishment of a medical consultation every 15 minutes as minimum productivity standard ^16^.

They are routines that diverge from what is recommended in official documents, since, when confronted with situations of violence, professionals need time to establish bonds that can contribute to children and adolescents being able to express their fears and anxieties. And time is also required for professionals to be able to assimilate situations and formulate a truly effective intervention plan in terms of protection. These are actions that will hardly be carried out in 5, 10 or 15 minutes. In this sense, the high demand for care may contribute to the insufficient recording of suspected or confirmed cases. In addition, sexual violence must be addressed - a factor that silences many children and adolescents due to shame and fear, making it difficult to verbalize the aggression and demand more attention from the professionals. Authors have demonstrated that the process of expressing having witnessed sexual violence is long and does not occur in a single event^17^.

Concerning the interplay that assigns the responsibility for the care of the victims of violence to a health service, or to a social service, it is necessary to question whether this is not a reflection of a true joint limitation of both social and health services, due to the multifaceted character of violence, which generates physical, psychological and social consequences^6^. Moreover, this interplay seems to be a reflection of the professionals' difficulty in taking responsibility for their care service and for reporting violence. Researchers point out that in the field of nursing violence against children and adolescents is often understood as a problem whose resolution is not part of the competencies of this professional group^18^.

Faced with these aspects, it is up to professionals and institutions to strengthen shared actions, establishing an effective protection network that includes reference and counter-reference services capable of acting on the multiple demands for attention and care expressed by victims and families in situations of violence.

## Conclusion

The professional practices, facing situations of intra-family violence against children and adolescents are organized based on the influence of power relations that permeate the institutional context, institutional routines managed in terms of demand and in the interplay between the conception of violence as a problem of public health and violence as a social problem. These institutional relationships end up making it difficult for professionals to incorporate and operationalize the protection guidelines established in official documents that refer to violence, contributing to the organization of practices that are not effective in terms of protection.

In view of these aspects, it is important to emphasize the importance of analyzing the institutional contexts and practices of professionals confronted with intra-family violence, in order to understand the subjacent order, and then to formulate strategies that allow to re-adjust the care provided.
